# Color Stability of Various Orthodontic Clear Aligner Systems after Submersion in Different Staining Beverages

**DOI:** 10.3390/ma17164009

**Published:** 2024-08-12

**Authors:** Nicolae Daniel Olteanu, Ionut Taraboanta, Tinela Panaite, Carina Balcos, Sorana Nicoleta Rosu, Raluca Maria Vieriu, Stefania Dinu, Irina Nicoleta Zetu

**Affiliations:** 1 Department of Oral and Maxillofacial Surgery, Faculty of Dental Medicine “Grigore T. Popa”, University of Medicine and Pharmacy from Iasi, Str. Universitatii 16, 700115 Iasi, Romania; daniel.olteanu@umfiasi.ro (N.D.O.); ionut-taraboanta@umfiasi.ro (I.T.); tinela-panaite@umfiasi.ro (T.P.); carina.balcos@umfiasi.ro (C.B.); raluca-maria.vieriu@umfiasi.ro (R.M.V.); irina.zetu@umfiasi.ro (I.N.Z.); 2 Department of Pedodontics, Faculty of Dental Medicine, “Victor Babes”, University of Medicine and Pharmacy Timisoara, No. 9, Revolutiei Bv., 300041 Timisoara, Romania; dinu.stefania@umft.ro

**Keywords:** clear aligner, polyethylene terephthalate glycol, thermoplastic elastomer, color stability, staining beverages

## Abstract

This study aimed to compare the color changes in two different orthodontic clear aligner systems after submersion in various beverages for 14 days. The tested aligner systems were Taglus Premium made of polyethylene terephthalate glycol (the TAG group) and CA^®^ Prodin+ made of a transparent copolyester and a thermoplastic elastomer (the PRO group). A total of 56 samples were firstly divided into two groups according to the tested system—TAG and PRO. Each group was subsequently divided in four subgroups according to immersion solution: A—artificial saliva, B—cola, C—coffee, D—red wine. Color measurements were performed on Days 1, 7 and 14 using a portable colorimeter and the CIE L*a*b* system. The obtained results showed significant color changes in both materials when exposed to coffee and red wine (*p* > 0.05). Samples in the PRO group showed a greater susceptibility to discoloration (higher ΔE values) when compared to the TAG group after submersion in cola (*p* = 0.025), coffee (*p* = 0.005) and red wine (*p* = 0.041) solutions. Statistical analysis revealed that all of the color parameters ΔL*, Δa*, Δb* and ΔE of both tested materials were affected by submersion in coffee solution for 14 days. In conclusion, the CA^®^ Pro+ aligner system is more prone to staining compared to the Taglus material after submersion for 14 days in cola, coffee and red wine solutions. Submersion for 14 days in coffee solution alters all of the color parameters (ΔL, Δa, Δb and ΔE) of both tested aligner materials.

## 1. Introduction

Currently, patients seeking orthodontic treatment demand not only high-quality results, but also a comfortable and aesthetic treatment experience [1]. The patients’ preference for esthetic dental treatments has led to the development of various orthodontic appliances that are as inconspicuous as possible and readily accepted by patients due to their clinical, esthetical and social comfort.

The recent improvements in computer-aided manufacturing/design (CAD/CAM) and the development of new dental materials have allowed the appearance of revolutionary orthodontic treatments [2]. These materials are composed of thermoplastic resin polymers such as polyvinyl chloride, polyethylene terephthalate, polyethylene terephthalate glycol and thermoplastic polyurethane [1,2].

The ideal orthodontic appliance has been described by Proffit et al.; therefore, it must be esthetic, lightweight, should not interfere with occlusion or hygiene, should not affect oral tissues, should be capable of withstanding masticatory forces, allow controlled forces to be applied between treatment sessions and provide good anchorage control [3]. The concept of transparent aligners was first introduced in 1946 as a tooth positioner manufactured by thermoplastic molding technology and used to generate minor tooth movements during the final stages of orthodontic treatment [4].

Although the initial main purpose of orthodontic therapy based on clear aligners was to address low and moderate dento-alveolar incongruence and to close small spaces, the technique has continuously evolved through the development of new materials used in the manufacture of aligners, dental attachments and special auxiliary systems, which now allow the approach of a wide range of malocclusions [1,4].

The color stability of aligners is crucial, as any discoloration may significantly affect their aesthetic value. Previous studies on thermoformed aligners have examined their resistance to staining in detail, attributing color stability to both material properties and manufacturing process [5,6]. Other research has shown that thermoformed aligners maintain their color when exposed to staining beverages such as coffee, tea and wine, due to the surface characteristics and chemical composition of the polyurethane [7].

However, through prolonged contact with acidic beverages, these materials might undergo surface wear, making them susceptible to discoloration. Also, an important role is played by the water absorption capacity of the materials, which leads to a hydrolytic degradation of the polymers, affecting the mechanical and aesthetic properties [8]. Moreover, the beverage temperature may cause the thermal expansion or contraction of these materials, increasing their susceptibility to deterioration and discoloration over time [9].

The current orthodontic therapy applied to both children and adults benefits from minimally invasive approaches as well as esthetic appliances, according to patients’ requirements. Patients’ current demands for a healthy smile have led to the development of new types of orthodontic appliances, such as invisible ones. In the last decade, orthodontic aligners have become more thin, transparent and comfortable to patients and at the same time, efficient in the therapy of several dento-maxillary anomalies [10].

In spite of their increasing popularity, investigations to assess the staining susceptibility of aligner materials are scarce [11]. Through this study, we propose to address this gap by following the behavior of aligners after a prolonged exposure to different coloring solutions. The study results will be likely to provide valuable insight on the durability and longevity of aligner materials under real-life conditions and offer guidance to clinicians and patients in taking decisions on the material selection and care of aligners.

The aim of this in vitro study was to compare the colorimetric changes in two materials used as transparent aligners after immersion for 14 days in different staining beverages (cola, coffee and red wine). Color measurements were performed after Days 1, 7 and 14. The null hypothesis of the study stated that no differences in the color of each of the two materials would be observed after submersion for 1, 7 and 14 days in red wine, cola and coffee solutions.

## 2. Materials and Methods

The study was conducted in accordance with the Declaration of Helsinki and the rules imposed by the Ethics Committee of “Grigore T. Popa” University of Medicine and Pharmacy of Iasi, Romania (Agreement No. 66/2021).

### 2.1. Sample Preparation

The sample size was calculated using G*Power software version 3.1. developed at the Heinrich-Heine University of Düsseldorf, Germany. An effect size of 0.5, an alpha value of 0.05 and a power of 0.8 were used. The obtained results indicated a minimum number of 34 samples to be used in the study.

**Figure 1:**
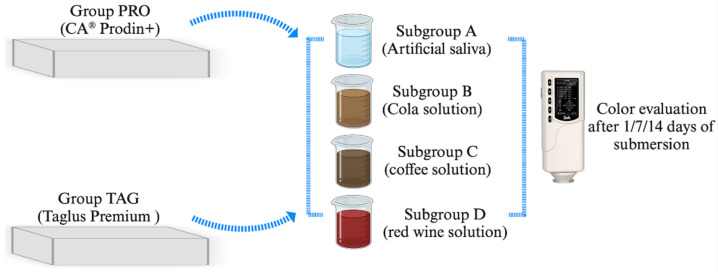
Study design and distribution of samples in groups and subgroups.

A total of 56 samples were used to perform this study. The samples were divided into 2 groups corresponding to the material from which they were made. Each group was then divided into 4 subgroups corresponding to the immersion solution. The distribution of the samples into groups and subgroups is shown in Figure 1.

The samples in the TAG group (*n* = 28) made of the Taglus Premium system (Taglus Company, Mumbai, India) contained polyethylene terephthalate glycol, while samples in the PRO group (*n* = 28) made of the CA^®^ Prodin+ system (SCHEU-DENTAL GmbH, Iserlohn, Germany) contained a transparent copolyester and a thermoplastic elastomer. The samples were realized by a thermoforming process using BIOSTAR^®^ (SCHEU-DENTAL GmbH, Iserlohn, Germany) with a 3D-printed mold obtained with a SprintRay 3D printer (SprintRay GmbH, Weiterstadt, Germany), thus resulting rectangular samples of 10 mm length/10 mm width/0.75 mm height. The samples were then kept in distilled water at 37 °C for 24 h in a Biobase incubator (Biobase BJPXH30II, Biodusty, Jinan, China).

### 2.2. Submersion in Coloring Solutions

The samples were then submersed in the following coloring solutions: Subgroup A—AFNOR artificial saliva (Biochemazone™, Leduc, AB, Canada) considered as a control subgroup; and three study subgroups: Subgroup B—Pepsi Cola, (PepsiCo. Inc., New York, NY, USA), Subgroup C—coffee Nescafe Brasero (Nestle, Vevey, Switzerland) and Subgroup D—dry red wine, 13.5% alcohol by volume, Negru de Purcari 2015 (Purcari Winery, Purcari, Republic of Moldova). AFNOR artificial saliva was composed of NaCI 6.7 g/L, KCl 1.2 g/L, NaHCO_3_ 1.5 g/L, NaH_2_PO_4_ H_2_O 0.26 g/L, KSCN 0.33 g/L and urea 1.35 g/L. Instant coffee was prepared by dissolving 3.6 g of coffee in 300 mL hot distilled water and filtered after 10 min. Samples were immersed in solution for 14 days and stored in an incubator at 37 °C. The coloring solutions were changed every 24 h. The pH values of the tested solutions were the following: artificial saliva—7.4; cola solution—2.71; coffee solution—5.33; red wine solution—3.85. The pH value was evaluated every 24 h using a portable pH meter (Thermo Scientific Eutech pH 5+, Vernon Hills, IL, USA).

### 2.3. Color Evaluation

The CIE L*a*b* system was used to determine the color variation in each study sample using a Precision Colorimeter NR10QC spectrophotometer (3NH Technology Co., Ltd., Shenzhen, China) with manual calibration and a 0.3 mm focused light beam.

Measurements were performed after 1, 7 and 14 days of submersion in the staining solutions. Subsequently, the samples were washed of residual dyes with distilled water and dried with the water–air spray from the dental unit. For color determination, samples were placed on a white sheet of paper to avoid color absorption. L*, a* and b* values were determined by a single operator and repeated three times for each sample. The mean of the three measurements was the value assigned to the sample. The color variation was calculated using the following formula:

ΔE = [(ΔL*)^2^ + (Δa*)^2^ + (Δb*)^2^] ½ ½ (11), where ΔE values represent the total color deviation, ΔL* is the brightness deviation, Δa* is the red–green axis deviation and Δb* is the yellow–blue axis deviation. The values were calculated according to the following formulas [12,13]:

Δa* = a*T − a*R (where T is the test solution; R the control solution—artificial saliva)

Δb* = b*T − b*R

ΔL* = L*T − L*R

### 2.4. Statistical Analysis

The obtained data were statistically analyzed using IBM SPSS software version 29.0.0. (IBM SPSS Inc., Chicago, IL, USA). The normality of data distribution was assessed using the Shapiro–Wilk test and the homogeneity of variances was verified with Levene’s test, while the differences between the groups and subgroups were analyzed using ANOVA one-way and Tukey post-hoc tests, independent samples *t* tests and the Kruskal–Wallis test. The significance level was set at 0.05.

## 3. Results

**Figure 2:**
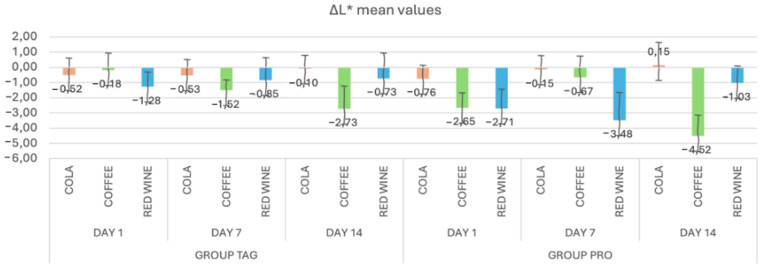
Mean values and standard deviations of the ΔL* parameter within each study stage for the TAG and PRO groups.

In Figure 2, the mean values and standard deviations of the ΔL* parameter within each study stage for the TAG and PRO groups are presented. It can be observed that in the TAG group, on Day 1 the highest value was recorded for the samples submersed in red wine solution (−1.28 ± 0.97), on Day 7 for the samples submersed in coffee solution (−1.52 ± 0.68) and on Day 14 also for the samples submersed in coffee (−2.73 ± 1.50). For the PRO group, on Day 1 the highest negative value was recorded for the samples submersed in red wine solution (−2.71 ± 1.26), on Day 7 for the samples submersed in red wine (−3.48 ± 1.82) and on Day 14 for the samples submersed in coffee (−4.52 ± 1.37).

Statistical analysis of the data within the TAG group shows that between the values obtained on Day 1 of submersion in coffee solution vs. Day 14 of submersion in the same solution, there were statistically significant differences in the parameter L* (*p* = 0.002). Within the PRO group, significant differences were recorded between the samples submersed in coffee solution for Day 1 vs. Day 7 (*p* = 0.023), Day 1 vs. Day 14 (*p* = 0.033) and Day 7 vs. Day 14 (*p* < 0.00), respectively, and between the samples submersed in red wine solution: Day 7 vs. Day 14 (*p* = 0.004).

**Figure 3:**
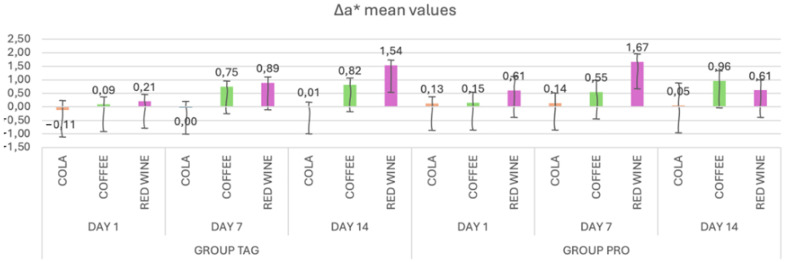
Mean values and standard deviations of the Δa* parameter within each study stage for the TAG and PRO groups.

In Figure 3, the mean values and standard deviations of the Δa* parameter within each subgroup of both the TAG and PRO groups are presented. Within the TAG group, on Day 1 the highest value was recorded for the samples submersed in red wine solution (0.21 ± 0.25), on Day 7 for the samples submersed in wine (0.89 ± 0.22) and on Day 14 also for the samples submersed in red wine (1.54 ± 0.19). As for the PRO group, on Day 1 the highest value was recorded for the samples submersed in red wine solution (0.61 ± 0.51), on Day 7 also for the samples submersed in wine (1.67 ± 0.28) and on Day 14 for the samples submersed in coffee (0.96 ± 0.38).

Statistical analysis of the data within the TAG group shows that there were significant differences within the samples submersed in coffee between Day 1 vs. Day 7 (*p* < 0.001) and Day 1 vs. Day 14 (*p* < 0.001), and between the samples submersed in wine: Day 1 vs. Day 7 (*p* = 0.029) and Day 1 vs. Day 14 (*p* < 0.001). In the PRO group, significant differences were recorded between the values obtained on Day 1 vs. Day 14 for the samples submersed in coffee solution (*p* = 0.016).

**Figure 4:**
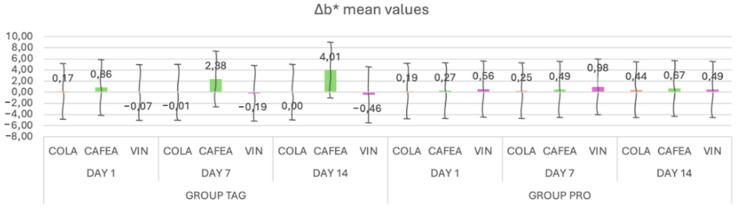
Mean values and standard deviations of the Δb* parameter within each study stage for the TAG and PRO groups.

When analyzing the mean values of the Δb* parameter (Figure 4) for the TAG group, it can be observed that on Day 1 the highest mean value was recorded for the samples submersed in coffee (0.86 ± 0.28), on Day 7 for the samples submersed in coffee (2.38 ± 0.23) and on Day 14 also for the samples submersed in coffee (4.01 ± 0.34). For the PRO group, on Day 1 the highest value was recorded for the samples submersed in wine (0.56 ± 0.07), on Day 7 for the samples submersed in coffee (0.98 ± 0.98) and on Day 14 for the samples submersed in coffee (0.67 ± 0.41).

The results of the statistical tests show that within the TAG group there were significant differences between the samples submersed in coffee on Day 1 vs. Day 7 (*p* < 0.001) and Day 1 vs. Day 14 (*p* < 0.001). For the PRO group, statistically significant differences were obtained between Day 1 vs. Day 14 (*p* = 0.00) for the samples submersed in coffee solution.

In Table 1, the mean values and standard deviations of the ΔE values within each study stage for TAG group are presented. It can be noted that on Day 1 the highest value was recorded for the samples submersed in coffee (1.43 ± 0.83), on Day 7 for the samples submersed in wine (2.96 ± 0.52) and on Day 14 for the samples submersed in coffee (5.06 ± 0.87).

Within the PRO group, the highest ΔE value on Day 1 was recorded for the samples submersed in wine (2.91 ± 1.11), on Day 7 for the samples submersed in wine (4.03 ± 1.49) and on Day 14 for the samples submersed in coffee (6.86 ± 0.66).

**Figure 5:**
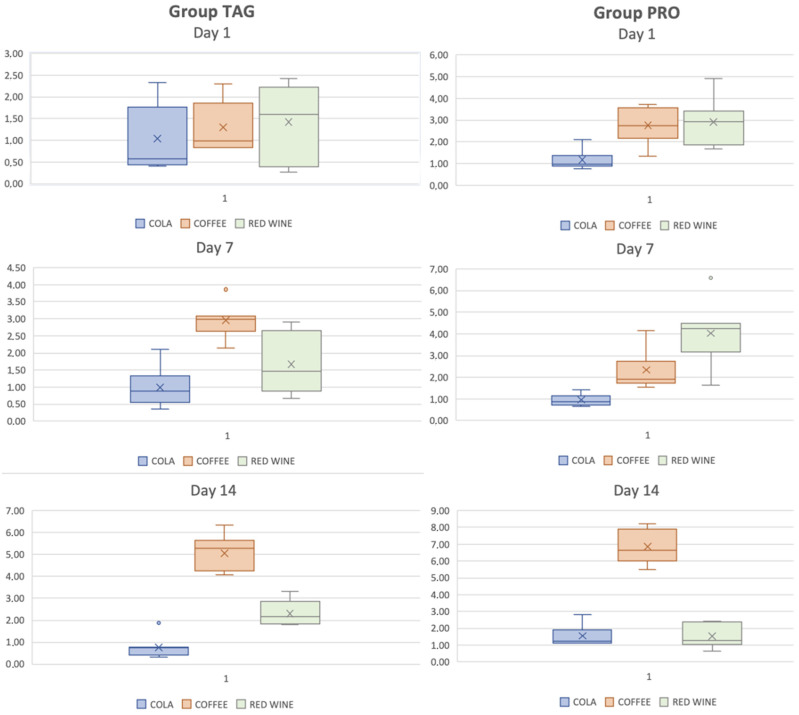
Box-plot representation of the distribution of ΔE values of each group and subgroup on each test day.

In Figure 5, box-plot graph is used to illustrate the distribution of the ΔE values of each group and subgroup by the end of each test day. The results of the statistical tests showed significant differences within the TAG group between the samples submersed in coffee on Day 1 vs. Day 7 (*p* < 0.001), Day 1 vs. Day 14 (*p* < 0.001) and Day 7 vs. Day 14 (*p* < 0.001). For the PRO group, statistically significant differences were obtained between the samples submersed in coffee solution between the values recorded on Day 1 vs. Day 14 (*p* < 0.001) and Day 7 vs. Day 14 (*p* < 0.001), and between the samples submersed in wine solution: Day 7 vs. Day 14 (*p* = 0.002).

Comparative statistical analysis of the ΔE values of the two tested materials showed differences between the values recorded for the samples submersed for 1 day in coffee solution (*p* = 0.003) and in wine (*p* = 0.015), and for those submersed for 7 days in wine solution (*p* = 0.004) and for the samples submersed for 14 days in cola solution (*p* = 0.025), coffee (*p* = 0.005) and wine (*p* = 0.041).

## 4. Discussion

This in vitro study is of significant applicability in clinical practice, as orthodontic therapy with aligners is increasingly used due to their esthetic and clinical advantages [14]. Most of the currently used aligners are thermoplastic polymers, polyurethanes and polyesters, such as those based on polyethylene terephthalate glycol [15].

Aligners are made of amorphous polymers such as polyurethane, polycarbonate and polyethylene terephthalate glycol that benefit from a highly transparent appearance, all three types of materials being widely used in dentistry, especially in orthodontics, due to their superior mechanical and aesthetic properties [9].

Polyurethane has excellent mechanical properties such as elasticity, flexibility, chemical resistance and ease of processing. These properties make it ideal for use in various dental appliances such as bruxism mouth guards, prosthodontic bases and other orthodontic appliances requiring flexibility and durability [16].

Polycarbonate is widely used in orthodontic appliances such as esthetic brackets and clear aligners because of its excellent optical, physical and chemical characteristics. Polycarbonate is preferred in dentistry because of its transparency, making it esthetically attractive to patients, as well as its impact resistance, thus ensuring durability in use [17].

Polyethylene terephthalate glycol (PET-G) is an amorphous co-polymer of PET that does not crystallize and is a relatively hard material with good mechanical properties, wear resistance and dimensional stability. In dentistry, it is commonly used to produce transparent aligners due to its resistance to deformation and ability to maintain a precise shape in the long term [16,18]. Although they are biocompatible materials, they still have some disadvantages related to dimensional instability, low resistance to wear and chewing forces, but also a change in esthetic properties determined by the absorption of water and of food colorants [6]. The polyethylene terephthalate glycol aligners are maintained in the oral cavity for a period of about 14 days, after which the clinical situation is assessed, and they are replaced with others until the final result is obtained. During this period, the food and drinks consumed by patients may lead to a loss of transparency. This phenomenon is explained by an increase in water absorption that may lead to the adsorption of food pigments [6,16].

In a study conducted in 2016 in which aligners made of different materials were tested and immersed for 14 days in coffee, black tea and red wine solutions, a higher color change was obtained in polyurethane aligners compared to polycarbonate or polyethylene terephthalate glycol. The authors explained the results by stating that the polyurethane material adsorbs pigments from colored beverages more than the other materials, because its water absorption capacity is higher and thus entrains the pigments in coffee and tea in particular. The roughness of the materials submersed in the three tested solutions was also analyzed in that study, and the authors observed that the same polyurethane material exhibited a rougher surface; thus, the surface condition of the materials may play an important role in enhancing the coloration [6].

Also, the pigmentation of the aligners may be influenced by the acidity of the food or drink ingested by the patients, the frequency of their consumption and the time they are kept in contact with the staining factors [19]. Our study consisted of the colorimetric evaluation after 1, 7 and 14 days of two new generation materials, composed of polyethylene terephthalate glycol and thermoplastic copolyesters and elastoplastic, which were immersed in different colored beverages (coffee, red wine and cola). The manufacturers of the polyurethane aligners recommend that patients not consume colored food or beverages while wearing the appliances.

The Taglus Premium material showed more pronounced changes in mean ΔL* values in samples submersed in red wine solution (−1.28 ± 0.97), followed by cola (−0.52 ± 1.12) and coffee (−0.18 ± 1.12). In contrast, in the CA^®^ Pro+ material, the changes in transparency after one day were higher for samples soaked in wine (−2.71 ± 1.26), followed by coffee (−2.65 ± 0.97) and cola (−0.76 ± 0.89).

Polyethylene terephthalate glycol materials did not show significant changes in transparency after one day of contact with staining solutions, due to their stable chemical composition and resistance to wear and chemical degradation in the oral environment [20]. The higher colorimetric values obtained for the CA^®^ Pro+ material can be explained by its water absorption capacity of 0.13% over 24 h, at a constant temperature of 23 °C. However, the samples evaluated by us were kept during the tests in an incubator at 37 °C, simulating the normal temperature in the oral cavity. The ΔE parameter, which represents the total color deviation, recorded the highest value on Day 1 for the samples submersed in coffee solution (1.43 ± 0.83), on Day 7 for the samples submersed in wine (2.96 ± 0.52) and on Day 14 for those submersed in coffee (1.24 ± 0.83). Our obtained results showed that the CA^®^ Pro+ material is more prone to staining compared to Taglus Premium after submersion for 1 day in coffee solution, while in red wine solution it showed significant changes for the whole tested period. Submersion for 14 days in coffee solution alters all of the color parameters (ΔL*, Δa*, Δb* and ΔE*) of both tested materials.

These results agree with another in vitro study which showed that polymers presented changes in transparency after 7 days of soaking in colored beverages [14]. The results of other studies have shown that thermomolding affects the transparency of the thicker material, decreasing it and increasing the water absorption properties, but may also alter the surface hardness. Thus, previous research also suggests that the thermomolding process decreases the thickness of the aligners compared to the initial size of the thermoplastic foil [21]. Specific conditions in the oral cavity, related to the salivary environment such as salivary enzymes, pH, temperature and the bacterial environment may negatively affect aligner transparency maintenance throughout the treatment.

Polyurethanes are susceptible to degradation over time through exposure to light, heat, moisture and enzymes. They can also exhibit oxidative degradation and absorb water if used for longer periods of time, leading to changes in physical and aesthetic properties [22]. The same conclusion as ours was reached by other studies that examined polyethylene terephthalate glycol or copolyester-based materials, stating that these materials compared to polyurethanes do not markedly change their transparency after submersion in staining beverages [11,23].

Three-dimensionally printed thermoplastic materials used as aligners can have acrylonitrile-butadiene-styrene, epoxy resins, polylactic acid, polyamide, glass-filled polyamide, silver, steel, titanium, photopolymers, wax and polycarbonate in their composition. Their choice is based on their excellent characteristics, which are essential for obtaining the desired clinical results and perfect adaptation to the teeth [9]. Their production process may influence the final thickness of the aligners, negatively affecting them if they are created by 3D printing, although this process is time-saving and produces stronger and more elastic aligners compared to conventionally thermoformed ones [17,24]. Other authors have stated that the variations in thickness do not affect the clinical efficiency and the thermoforming process does not alter the active or passive configurations of these appliances [25,26].

The colorimetric variation in the aligners tested in our study can be explained by the fact that the tested dry red wine contained a variety of pigments, the most relevant of which are anthocyanins and tannins. Anthocyanins are the main pigments that confer red wine with its distinctive color. Anthocyanins are flavonoids and can range in color from red to blue, depending on the wine’s pH. Tannins, on the other hand, contribute to the structure and color of red wine. They play a role in wine color stability by interacting with anthocyanins and form polymerized pigments that are responsible for a more stable and deeper color of red wine as it ages. Other pigments are flavonols, such as quercetin and kaempferol, which although less abundant, contribute to the color and antioxidant characteristics of the wine. Overall, the combination of these pigments and their complex interactions confer an unique color and chromatic stability to dry red wine [13,27].

Coffee contains slow-release low-polarity yellow pigments that penetrate organic substrates. Therefore, significant changes were observed in both groups for the samples submersed in coffee for 1 day, these results being in agreement with other studies [6,19].

The other tested staining solution was Pepsi Cola that contains caramel as the main colorant. This is an artificial pigment obtained by heating sugar and is often used in carbonated drinks to confer the characteristic brown color. The pigment-impregnating capacity of the solutions in the study is also determined by their pH value. The increased acidity of the solutions may lead to surface changes in the tested materials by producing chemical and physical alterations of the surface through acid wear [20,28,29]. Both pigments and the pH of the solutions play a major role in the color changes in the materials used for alignment [30]. The pigments in coffee, tannins in red wine, or caramel in cola drinks adhere easily to the surface of clear aligners producing visible color changes. On the other hand, an acidic environment may increase the susceptibility to staining due to altered properties and an enhanced surface roughness of the material that attracts the retention of colorants. Thus, pigments color the surfaces whereas the presence of an acidic pH enhances the coloring effect [30,31].

The results of our in vitro study demonstrated that the inappropriate use of aligners during mealtimes can lead to a loss of transparency, as colorants in food and beverages can affect the color stability of thermoplastic materials [20,32]. Thus, orthodontists should advise patients who are concerned about esthetics to be aware of the possibility of visible changes in the color of aligners during use, these changes being closely related to their diet, hygiene habits and the fact that they have to remove these devices from their oral cavity during meals [12,19,33,34,35].

The results of the study reject the null hypothesis that there are no statistically significant differences between the two study materials submersed in the coloring solutions during the evaluation period. The limitations of the conducted study were that we evaluated only two materials from the same category of transparent aligners, namely polyethylene terephthalate glycol, at the same thickness of 0.75 mm and we did not use models on the teeth, which by their shape may influence the results of the physical and chemical tests.

However, to justify the conclusions of the study, further in vitro and in vivo studies are needed using other evaluation methods, such as scaling electron microscopy, microhardness and wear resistance analysis of these materials after immersion in colored solutions, to validate the results obtained.

For greater clinical relevance, future in vitro studies should be able to reproduce oral environmental conditions, such as temperature and pH variations, enzymatic and microbial activity and the mechanics of masticatory movements.

## 5. Conclusions

The CA^®^ Pro+ aligner system is more prone to staining compared to the Taglus material after submersion for 14 days in cola, coffee and red wine solutions.

Submersion for 14 days in coffee solution alters all of the color parameters (ΔL, Δa, Δb and ΔE) of the CA^®^ Pro+ and Taglus Premium aligner systems.

Immersion for 1, 7 or 14 days in red wine solution induced differences in the color stability of the two tested materials.

In order to obtain optimal esthetic results during the treatment period, which is usually 14 days, patients must follow the orthodontists’ instructions regarding diet, the consumption of coloring beverages and oral hygiene.

## Figures and Tables

**Table 1:** Mean values, standard deviations of ΔE and statistically significant differences between groups.

	Day 1			Day 7			Day 14		
	Cola	Coffee	Red Wine	Cola	Coffee	Red Wine	Cola	Coffee	Red Wine
TAG Group	−0.52 ± 1.12	−0.18 ± 1.12	−1.28 ± 0.97	−0.53 ± 1.03	−1.52 ± 0.68	−0.85 ± 1.48	−0.10 ± 0.90	−2.73 ± 1.50	−0.73 ± 0.89
PRO Group	−0.76 ± 0.89	−2.65 ± 0.97	−2.71 ± 1.26	−0.15 ± 0.92	−0.67 ± 1.41	−3.48 ± 1.82	0.15 ± 1.48	−4.52 ± 1.37	−1.03 ± 1.11
*p* values	**	* 0.003	* 0.015	**	**	* 0.004	* 0.025	* 0.005	* 0.041

* Statistically significant differences. ** Statistically non-significant differences.

## Data Availability

The data presented in this study are available from the corresponding author upon reasonable request.
